# Range Expansion of the Giant Water Bug *Lethocerus patruelis* (Stål, 1854) in Europe

**DOI:** 10.1002/ece3.72458

**Published:** 2025-11-12

**Authors:** Andrea Simoncini, Filippo Tomasi, Gentile Francesco Ficetola, Elia Lo Parrino

**Affiliations:** ^1^ Department of Environmental Science and Policy University of Milan Milano Italy; ^2^ Museo di Storia Naturale del Salento Lecce Italy; ^3^ University Grenoble Alpes Grenoble France

**Keywords:** climate change, Hemiptera, MaxEnt, niche dynamics, suitability models

## Abstract

Climate change is altering freshwater ecosystems, causing extinctions, range expansions, and facilitating biological invasions. Colonization by novel species can drastically affect local biodiversity, particularly in aquatic habitats. *Lethocerus patruelis* (Stål, 1854) is a large predatory aquatic insect whose distribution spans from the Balkans to south‐eastern Asia. In the last decades, *L. patruelis* sightings outside its known range have increased, particularly in Italy. The aim of this study was to assess the drivers of this expansion and the potential for future spread. We collected records of *L. patruelis* using published literature, citizen‐science platforms, and social media. These data were used to test for directional expansion and to compare historical and novel niches. Second, we used observations from the historical range of the species to create a suitability model using MaxEnt, testing it using observations from Italy. Finally, we projected the model under three future climatic scenarios to assess the potential for future expansions. We detected a significant westward and southward expansion of *L. patruelis* in Italy. Niche conservatism between historical and novel ranges was observed. Nonetheless, we found limited overlap and a high level of niche unfilling, suggesting an ongoing colonization process. The suitability model showed good predictive performance, indicating a preference toward Mediterranean climates and a selection against agricultural areas. Suitable areas were predicted to increase under all three future climatic scenarios. This study suggests an ongoing spread of *L. patruelis* and a strong expansion potential in Europe facilitated by climate change.

## Graphical abstract

We modeled the expansion of the giant water bug, with a focus on niche dynamics and habitat suitability. Our results show evidence for an ongoing expansion, with strong potential for further spread under future climatic conditions.

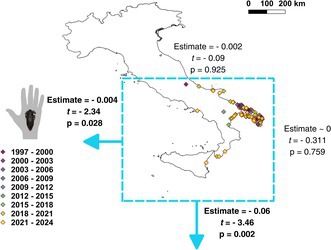

## 1 Introduction

Global changes are shaping the biodiversity of insects worldwide (Raven and Wagner 2021; Wilson and Fox 2021; Outhwaite et al. 2022). Climate change, land use transformation, global transportation, and other anthropogenic drivers are altering insect behavior, life histories, distributions, and abundances, leading to community restructuring with often unpredictable ecological consequences (Yang et al. 2021). Aquatic insects are generally more vulnerable to global change than their terrestrial counterparts, owing to their narrower ecological niches and lower tolerance to variations in both abiotic and biotic conditions (Harvey et al. 2023). In addition, their habitats, such as ponds and rivers, are increasingly threatened by water scarcity, whose frequency and severity are rising in some areas due to climate change (Trenberth et al. 2014; Spinoni et al. 2017).

The giant water bug *Lethocerus patruelis* (Stål, 1854) is a large aquatic hemipteran distributed from Southern Europe to Southeast Asia (Perez and Goodwyn 2006; Sareein et al. 2019). It is the largest true bug in Europe, reaching body lengths of 6–8 cm (Perez and Goodwyn 2006). *L. patruelis* and related species are predatory insects that primarily feed on vertebrates, including fish, amphibians, and reptiles (Ohba 2019, 2011; Christopoulos et al. 2022). As in other members of the family Belostomatidae, males exhibit marked parental care by guarding the eggs until they hatch (Ohba 2019). Despite its considerable size and dispersal capability, *L. patruelis* remains infrequently recorded in Europe. Most records come from social media, online news sources, or citizen science platforms, and its actual distribution is still poorly known (Corsini‐Foka et al. 2019; Davranoglou and Karaouzas 2021; Lo Parrino and Tomasi 2021; Cianferoni and Mazza 2023).

Over the past decades, the range of *Lethocerus patruelis* has expanded both northward and westward (Cianferoni and Nardi 2013; Grozeva et al. 2013; Stoianova and Simov 2016; Lo Parrino 2019). The species has been reported in Italy since 1997, and records now span across much of Southern Italy (Cianferoni and Nardi 2013; Lo Parrino 2019; Castiglione et al. 2021; Lo Parrino and Tomasi 2021; Cianferoni and Mazza 2023; Cianferoni et al. 2024). Observations of both sexes, along with the frequency and geographical spread of recent records, suggest a stable presence in the region, although a fully naturalized population has yet to be confirmed (Lo Parrino and Tomasi 2021; Cianferoni and Mazza 2023; Cianferoni et al. 2024). The origin of this presence remains uncertain: it is unclear whether it results from a natural range expansion or a human‐mediated introduction. The species was first detected near major Adriatic ports, and, due to its strong positive phototropism, it has been hypothesized that individuals may have arrived from the Balkans, following artificial lights from ships and boats (Cianferoni and Nardi 2013). Based on this, the authors suggested that *L. patruelis* be considered an alien species in Italy. While this interpretation is still debated, it is important to note that both biological invasions and rapid natural expansions are often driven by similar mechanisms and can have comparable ecological impacts (Hoffmann and Courchamp 2016). As such, evaluating the potential spread of expanding species remains relevant, regardless of their origin, be it anthropogenic or natural.

Although invasive insects account for a considerable proportion of all invasive species globally, aquatic insects rarely become invasive (Fenoglio et al. 2016; Sendek et al. 2022). Nevertheless, invasive aquatic predators can profoundly alter food web dynamics in newly colonized freshwater ecosystems (Ficetola et al. 2012). Beyond their ecological impact, the spread of *Lethocerus patruelis* may also have implications for human health. Some Belostomatidae species have been suspected to act as potential vectors of pathogenic bacteria, such as *Mycobacterium ulcerans* (Haddad et al. 2010; Marion et al. 2010). Although no direct evidence of pathogen transmission by *L. patruelis* exists to date, at least one human bite incident has been reported (Cianferoni and Nardi 2013). For these reasons, the expansion of this species in Italy represents a compelling case study from both ecological and public health perspectives.

The aim of this study is to investigate the ongoing spread of *L. patruelis* in Italy, analyze its niche dynamics, and model its potential distribution under different climate change scenarios across Europe. This assessment may support the early detection and monitoring of a rapidly expanding species with the potential to disrupt freshwater food webs, with significant consequences for the conservation of vulnerable aquatic ecosystems. We hypothesize that *L. patruelis* is undergoing a continued range expansion in Italy, characterized by directional spread and a notable degree of niche unfilling and, possibly, expansion. Furthermore, we expect its potential distribution to increase under future climate scenarios.

## 2 Methods

### 2.1 Species Occurrences

Citizen science represents an important tool to track colonizing species, as it can generate high volumes of data across large areas (Silvertown 2009; Crall et al. 2010; Larson et al. 2020). Italian observations were collected through a systematic review of the literature and regular screening of online platforms and social media, as they can provide relevant information to assess the distribution of this species (Lo Parrino and Tomasi 2021; Davranoglou and Karaouzas 2021). Concerning online data, only occurrences associated with photographic evidence and detailed location descriptions were retained for analysis. In addition, a search was conducted on the Global Biodiversity Information Facility (GBIF, https://www.gbif.org/) in July 2024 to retrieve all available records of the species across its entire range. Observations with spatial uncertainty greater than 1 km were excluded. The resulting dataset was then merged with the curated Italian records.

Closely related species may exhibit distinct ecological responses to the same environmental conditions (Caro et al. 2005; Dalpasso et al. 2022), making it essential to avoid including misidentified records in distribution analyses. The dimensions and morphology of *Lethocerus* make representatives of this genus easy to recognize, but distinguishing *L. patruelis* from similar taxa can be challenging without careful examination of subtle morphological traits (Perez and Goodwyn 2006; Castiglione et al. 2021). Nonetheless, *Lethocerus patruelis* is the only representative of its family found in Europe and the Middle East (Perez and Goodwyn 2006; Grozeva et al. 2013; Sareein et al. 2019), and thus misidentification is extremely unlikely. Within Belostomatidae, *Lethocerus cordofanus* is the only other species known to occur in the Mediterranean basin, reaching the region through the Nile River (Perez and Goodwyn 2006). However, given the considerable geographical distance between the range of *L. cordofanus* and our study area, their co‐occurrence is unlikely (Castiglione et al. 2021) and has never been reported. Concerning Italian specimens, recent morphological and molecular analyses have consistently confirmed their identity as *L. patruelis* (Cianferoni and Nardi 2013; Cianferoni and Mazza 2023; Lamanna and Dima 2024; Raele et al. 2025). Therefore, the records from Europe and the Middle East can be safely attributed to *L. patruelis* . In the easternmost portion of its range, *L. patruelis* overlaps with the congener *L. indicus* (Nesemann and Sharma 2013; Sareein et al. 2019). The two species can be distinguished by dorsal light stripe patterns (Perez and Goodwyn 2006; Sareein et al. 2019), but their overall morphological similarity does not entirely preclude the risk of misidentification. To account for this, analyses were conducted twice: (i) using the full known distribution of *L. patruelis* , and (ii) excluding all records east of longitude 66°, where its range overlaps with that of *L. indicus* .

All subsequent analyses were performed using a 1 km grid resolution. To minimize spatial sampling bias, records located within 1.5 km of each other were spatially thinned using the thin function from the *spThin* R package (Aiello‐Lammens et al. 2015; Brown and Carnaval 2019), following best practices for bias reduction in species distribution modeling (Vollering et al. 2019). The final dataset consisted of 231, spanning from 1997 to 2024. Of these, 142 came from published literature and social networks (106 of which were from Italy) and 89 from GBIF. After applying spatial filtering procedures, 146 records were retained: 38 from Italy and 108 from the species' historical range. When excluding the easternmost portion of the range, where *Lethocerus patruelis* overlaps with *L. indicus* , the filtered dataset was reduced to 130 occurrences.

### 2.2 Environmental Variables

We adopted an expert‐based approach to perform an a priori selection of environmental variables to include in the analyses, following Santini et al. (2021). The selection was informed by a review of the available literature on the ecology of giant water bugs and aimed at identifying key factors potentially driving the species' expansion and range. The chosen variables fell into two categories: climate and land use/land cover (LULC).

Two bioclimatic variables were selected based on their ecological relevance for the species: temperature of the warmest quarter (bio10) and precipitation seasonality (bio15). The first was chosen due to its importance in facilitating dispersal, as species in the Lethocerinae subfamily require high temperatures to warm their flight muscles before taking off (Ohba 2019), and because most observations occur during summer months (Lo Parrino and Tomasi 2021). Precipitation seasonality (bio15) was included as a proxy for Mediterranean climatic conditions, which the species appears to prefer (Lo Parrino and Tomasi 2021). Climatic data for the period 1981–2010 were retrieved from the CHELSA database (Karger et al. 2017). While temporal ranges of occurrence data and climatic data do not perfectly align, we decided to use CHELSA because of the lack of better high‐resolution, temporally matched climatic datasets. Moreover, CHELSA represents long‐term 30‐year climatic averages, which correspond to the climatic conditions that organisms typically respond to. Such an approach is justified by the relative stability of species' geographic distributions over decadal timescales, which allows some flexibility in temporal mismatches between occurrences and environmental data (Feng et al. 2024).

For future projections (2041–2070), we used CHELSA CMIP6 climate scenarios under three Shared Socioeconomic Pathway–Representative Concentration Pathway (SSP–RCP) combinations: SSP1–RCP2.6 (sustainability pathway), SSP3–RCP7.0 (regional rivalry), and SSP5–RCP8.5 (fossil‐fuelled development). To account for variability across General Circulation Models (GCMs) and avoid model‐specific bias (Herger, et al. 2018), we used five GCMs: GFDL‐ESM4 (Dunne, et al. 2020), IPSL‐CM6A‐LR (Boucher, et al. 2020), MPI‐ESM1‐2‐HR (Müller, et al. 2018), MRI‐ESM2‐0 (Yukimoto et al. 2019), and UKESM1‐0‐LL (Tang et al. 2019).

LULC were characterized using three variables: percentage cover of trees, percentage cover of croplands, and percentage cover of freshwater habitats. Tree cover was included because forested areas are thought to provide suitable shelter for giant water bugs during winter, when individuals take refuge under leaf litter (Ohba 2019). Cropland surface was considered due to observations of *Lethocerus patruelis* in rice fields and irrigation channels (Lo Parrino and Tomasi 2021; Christopoulos et al. 2022). LULC data were derived from Lo Parrino et al. (2025), specifically the 1‐km resolution layers “Tree cover,” “Cropland,” and the combined cover of “Permanent water bodies” and “Herbaceous wetland” to represent freshwater habitats.

### 2.3 Testing for Directionality of Expansion

To assess whether *Lethocerus patruelis* is expanding its range in Italy and to determine the potential direction of this expansion, we fitted linear models relating the species' maximum and minimum latitude and longitude to the year of observation. All Italian records (*n* = 106) were included in the analysis, regardless of positional accuracy or spatial filtering. Residual plots were visually inspected to assess normality assumptions. All models met the assumption of normality, except for the one based on maximum longitude (easternmost records), for which a log transformation was applied to improve residual distribution. The slope coefficients of each model were interpreted as proxies for the rate and direction of range shift along the corresponding geographical axis. This approach provides a preliminary assessment of potential expansion trends and directions. Given the stochastic nature of citizen science data, it is considered unlikely that any directional pattern observed would stem solely from biased sampling effort or increased interest in specific regions.

### 2.4 Niche Dynamics

To assess the potential range expansion of the giant water bug in Italy, we estimated niche dynamics by treating Italy as the novel range and the rest of the species' distribution as the historical range, using the “*ecospat*” R package v. 4.1.0 (Di Cola et al. 2017). Six environmental variables were selected to characterize the species' niche: temperature of the warmest quarter and precipitation seasonality (from CHELSA, 1981–2010), and three land‐use/land‐cover (LULC) variables: tree cover, cropland cover, and aquatic habitats cover. To reduce dimensionality and multicollinearity among variables, we performed a principal component analysis (PCA). Environmental backgrounds were defined by extracting the centroids of 1 km resolution raster cells within a 100 km buffer surrounding occurrence points in both the historical and novel ranges, excluding cells where the species was present. Niche overlap between historical and novel ranges was quantified using two complementary metrics: Schoener's *D* and Hellinger's *I* (Warren et al. 2008; Broennimann, et al. 2012), both ranging from 0 to 1, with higher values indicating greater niche similarity. Further, we quantified niche dynamics through three metrics (Petitpierre, et al. 2012; Guisan, et al. 2014): (i) Niche unfilling (U): proportion of environmental conditions present in the historical range but absent in the novel range; (ii) Niche stability (S): proportion of shared environmental conditions between historical and novel niches; (iii) Niche expansion (E): proportion of environmental conditions unique to the novel range. These metrics were calculated based on the 10th quantile intersection of environmental densities in both historical and novel ranges, following Lo Parrino et al. (2023).

The significance of the niche metrics was assessed through niche equivalency and similarity tests, each performed with 100 replicates (Broennimann, et al. 2012). The equivalency test involves pooling all occurrences and randomly reallocating them into two datasets of the same sizes as the original historical and novel ranges; the niche overlap metric is then recalculated for each replicate to generate a null distribution. This test evaluates whether the observed niche overlap is more or less equivalent than expected if the two niches were drawn from the same underlying distribution. The similarity test, on the other hand, compares the observed historical niche with niches generated by randomly shifting the occurrence densities in the novel range. This assesses whether the observed niche overlap is more or less similar than expected by chance, given the environmental background of the novel range. A significant equivalency test indicates that the historical and novel niches differ more (or less) than would be expected if they were identical, while a significant similarity test indicates that the niches are more (or less) similar than expected given the available environments. Given that we tested for niche expansion, we expected that overlap and stability metrics would be significantly lower than expected by chance, while niche expansion and unfilling would be significantly higher under both tests. To confirm the robustness of our results, niche dynamics were re‐evaluated after excluding records from the easternmost portion of the species' range, where its distribution overlaps the distribution of *L. indicus* .

### 2.5 Species Distribution Modeling

Species distribution models (SDMs) relate species occurrences and pseudo‐absence or background points to environmental variables to estimate species distributions in both environmental and geographic space (Elith and Leathwick 2009). Once fitted, SDMs can be used to predict habitat suitability under current and future conditions, thereby assessing the potential impact of environmental change on species distributions (Elith and Leathwick 2009). In this study, we employed spatially explicit predictions from SDMs to visualize areas potentially suitable for the giant water bug under present conditions and various future climate scenarios. The presence of extensive suitable but currently unoccupied areas would further suggest that the species is not at equilibrium and is undergoing range expansion (Uden, et al. 2015).

We developed MaxEnt SDMs (Phillips et al. 2006) using thinned occurrence data from both the historical and novel ranges. MaxEnt is widely regarded as one of the best‐performing algorithms for SDMs and allows flexible tuning of hyperparameters to improve model reliability (Elith, et al. 2011; Valavi et al. 2023). To avoid collinearity issues, predictor variables were tested for correlation using Pearson's correlation coefficient, with values ≥ |0.7| indicating strong correlation and leading to the exclusion of one variable from each highly correlated pair (Dormann, et al. 2013). We performed this test on the predictor variables within the training area, defined as a 100 km buffer around all thinned occurrences, using the *pairs* function from the “raster” R package (Hijmans et al. 2015). No pairwise correlation exceeded 0.7, with the highest correlation observed between the two climatic variables (Figure S2.1). Additionally, we calculated the variance inflation factor (VIF) for each predictor to assess multicollinearity beyond pairwise correlations, confirming that all variables had VIF values below the recommended threshold of 3 (Zuur et al. 2010). Based on these results, all five predictors were retained for the MaxEnt modeling. Permutation importance for each predictor in the best‐performing MaxEnt model was calculated as the percentage decrease in model predictive performance when the predictor was permuted, providing insight into its relative contribution (Elith, et al. 2005). Response curves illustrating the effect of each predictor on habitat suitability were also generated. Background points (*n* = 20,000) were randomly sampled within a 100 km buffer around presence points to restrict sampling to areas accessible to the species (VanDerWal, et al. 2009). Given the giant water bug's strong attraction to artificial lights (Cianferoni and Nardi 2013), we anticipated sampling bias toward urbanized areas. To mitigate this bias, background points were sampled with a probability proportional to human population density, ensuring that background sampling reflected the same spatial bias as occurrence data, thereby improving model performance (Stolar and Nielsen 2015).

To evaluate model robustness and transferability, training and test datasets were geographically separated (Sutton and Martin 2022): occurrences outside Italy (*n* = 108, ~74%) were used for training, while occurrences within Italy (*n* = 38, ~26%) served as the test dataset. We tested eight values of the regularization multiplier (beta), ranging from 1 to 8 in increments of 1, and two feature combinations: LQ (linear and quadratic) and LQH (linear, quadratic, and hinge). Model performance was evaluated using the area under the receiver operating characteristic curve (AUC; Fielding and Bell 1997), a threshold‐independent metric, and the Continuous Boyce Index (CBI; Hirzel, et al. 2006). Both metrics range from 0 to 1, with values above 0.5 indicating predictive performance better than random on the Italian occurrence data. The model exhibiting the best balance between these two metrics was selected to generate spatial predictions of habitat suitability for the species across the European continent. Future projections were produced for the three SSP‐RCP scenarios described above, while keeping all non‐climatic variables constant at present‐day values due to the limited availability of future land‐use and land‐cover (LULC) data (Stanton, et al. 2012). For each SSP‐RCP combination, projections derived from different general circulation models (GCMs) were averaged, and the standard deviation among GCMs was calculated to assess prediction robustness. This entire modeling and projection process was repeated excluding records from the easternmost part of the species' range to verify the consistency of the results.

## 3 Results

**FIGURE 1:**
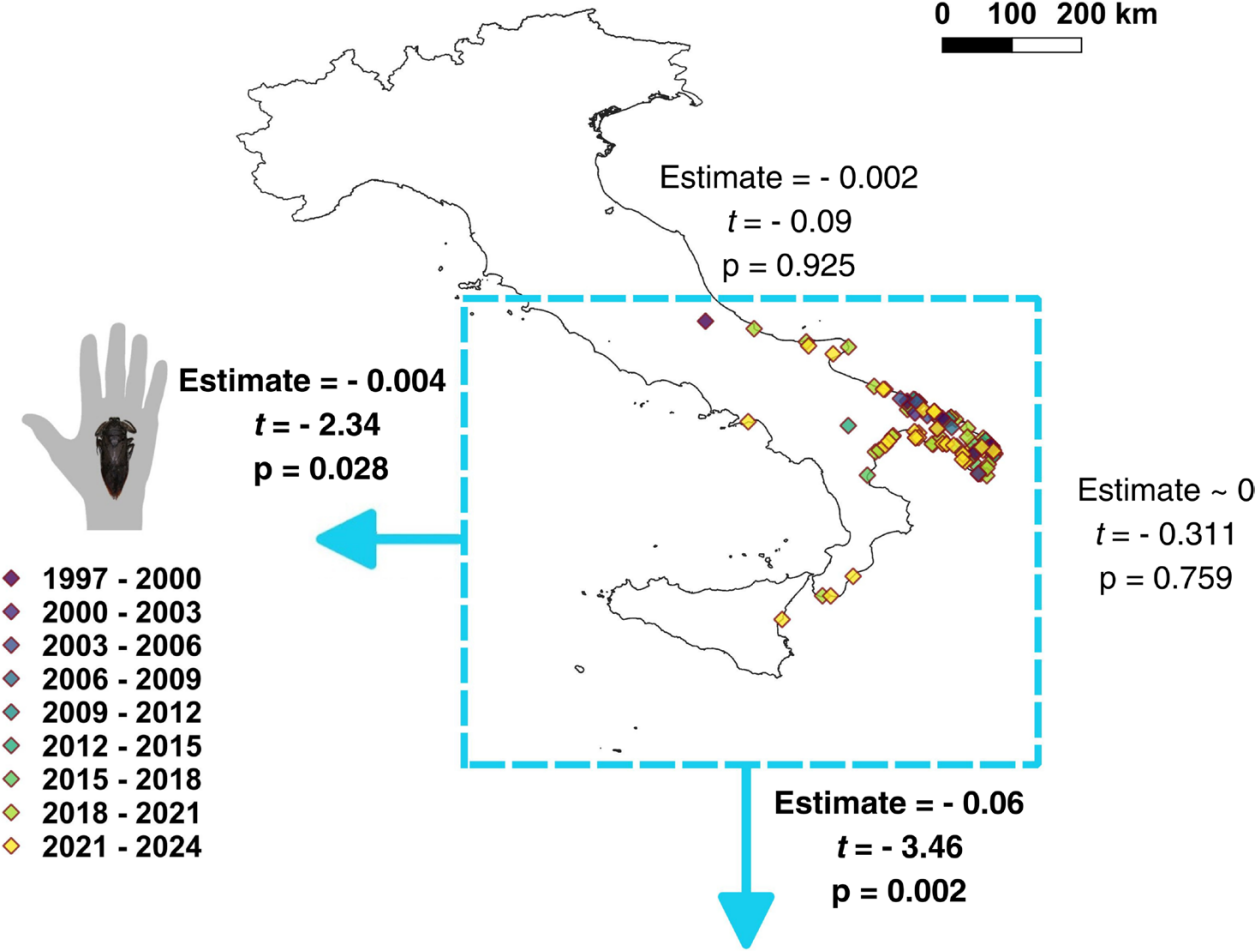
Observations of the giant water bug *Lethocerus patruelis* in Italy, along with the results of the directionality analysis of its range expansion. Four linear models were fitted separately for each range margin (north, south, east, west). Statistically significant trends are highlighted in bold and illustrated with arrows.

**FIGURE 2:**
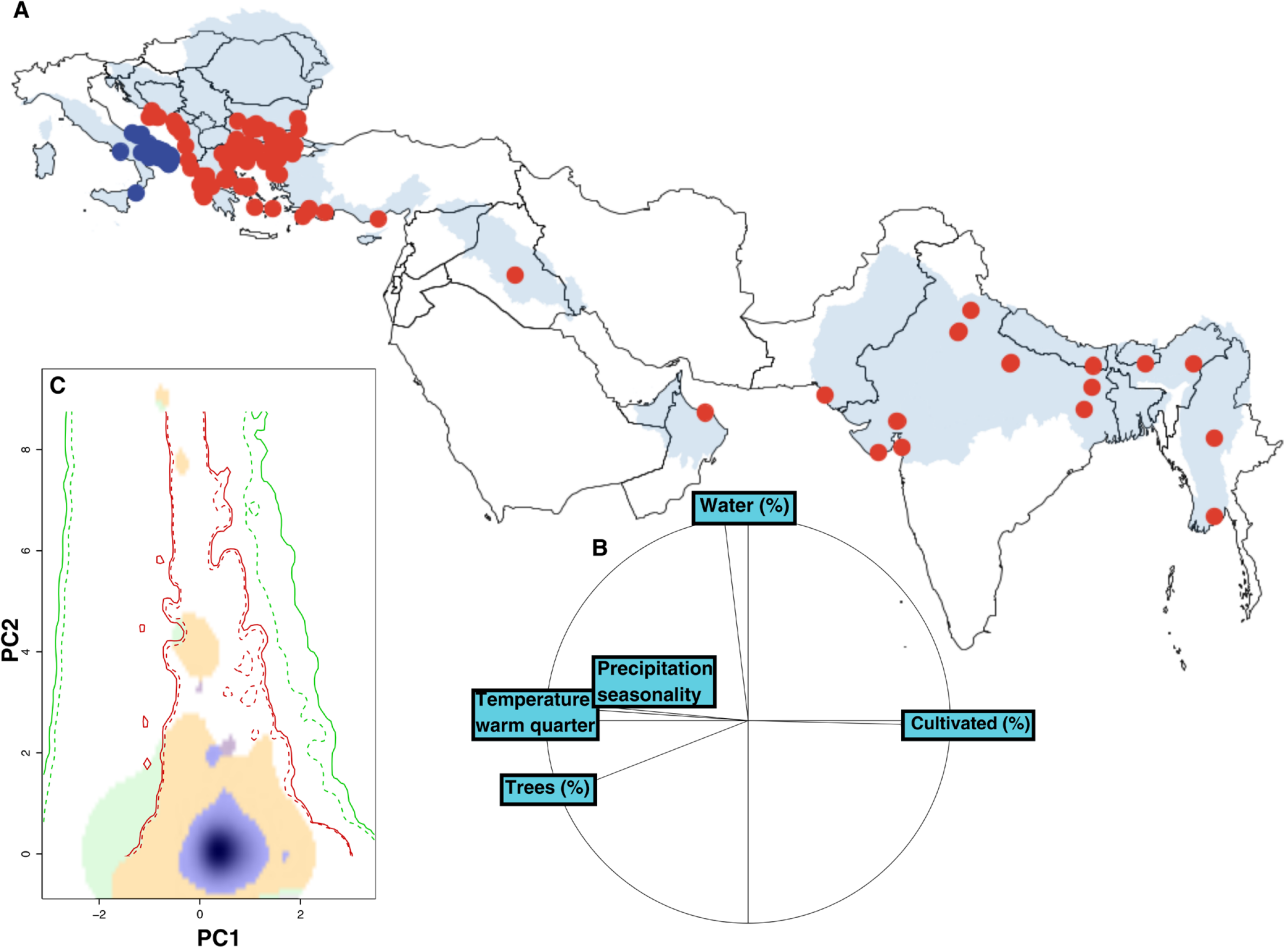
(A) Map of giant water bug *Lethocerus patruelis* occurrences in Italy: Historical range shown in red, novel range in blue. Shaded areas correspond to freshwater ecoregions of the world (Abell et al. 2008), and black lines indicate national borders. (B) Circular plot showing the contribution of each environmental variable used in the Principal Component Analysis. (C) Comparison of environmental niches between historical (green) and novel (red) ranges. The plot is based on the first two axes of the PCA‐env, summarizing the main environmental gradients. Solid contour lines show the full extent of environmental conditions for each range (green = historical, red = novel), while dashed lines indicate the 10th‐quantile intersection of environmental densities for each range. Colored areas represent niche dynamics: Blue = stability (conditions occupied in both ranges), orange = unfilling (conditions occupied in the historical but not in the novel range), and purple = expansion (conditions occupied in the novel but not in the historical range).

Linear models revealed a significant temporal shift in the southern and western boundaries of the species' novel range in Italy (Western boundary: estimate = −0.004, *p* = 0.028; Southern boundary: estimate = −0.061, *p* = 0.002), whereas no significant trends were observed for the northern and eastern limits (Figure 1). The first two axes of the PCA‐env explained 70.14% of variance (Data S1.1). Correlations between the environmental variables and PCA‐env axes are available as Supporting Information (Data S1.2). Niche metrics indicated a generally low overlap between the historical and novel niches. Niche equivalency tests were significant for both overlap indices (Schoener's SD = 0.104, *p* = 0.010; Hellinger's *I* = 0.260, *p* = 0.040; Table 1), suggesting that observed niche overlap was lower than expected by chance. This pattern was mainly driven by a high degree of niche unfilling in the novel range (*U* = 0.391, *p* = 0.010; Table 1), whereas niche expansion was negligible (*E* = 0.008; Table 1). Niche similarity tests did not detect significant differences between observed niche dynamics and those generated by comparing the historical niche with random niches drawn from the environmental space of the novel range, likely because environmental conditions in the novel range represent a subset of those available in the historical range (Figure 2). Results were consistent after excluding the easternmost occurrences, though niche overlap was lower (Schoener's SD = 0.018; Hellinger's *I* = 0.127) and niche unfilling higher (*U* = 0.600). Additional details on the results of niche dynamic tests can be found as Supporting Information (Data S1.3).

**TABLE 1:** Niche metrics estimated with the ‘ecospat’ R package (Di Cola et al. 2017) and obtained by comparing the historical and novel niche of the giant water bug *Lethocerus patruelis* , where the novel range is found in Italy and the historical range stretches from the Balkans to South‐eastern Asia.

Measure	Estimate	Equivalency *p*	Similarity *p*
Schoener's *D*	0.104	< 0.01***	0.683
Hellinger's *I*	0.260	0.040*	0.663
Expansion	0.008	0.614	0.386
Stability	0.992	0.614	0.386
Unfilling	0.391	< 0.01**	1

*Note:* Equivalency *p* = *p* value from a niche equivalency test, Similarity *p* = *p* value from a niche similarity test. Significance codes: *p ≤ 0.05; **p ≤ 0.01; ***p ≤ 0.001.

**FIGURE 3:**
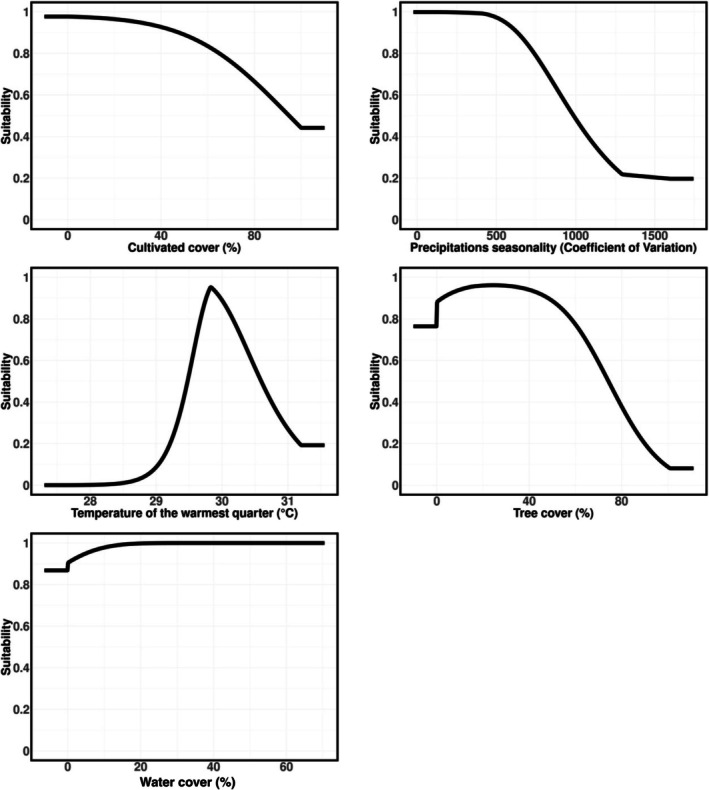
Response curves showing the effect of each predictor variable on habitat suitability for the giant water bug *Lethocerus patruelis* . The *y*‐axis represents habitat suitability, ranging from 0 (unsuitable) to 1 (highly suitable). Curves were derived from a fine‐tuned Maxent species distribution model, illustrating how suitability changes along the gradient of each environmental predictor.

**FIGURE 4:**
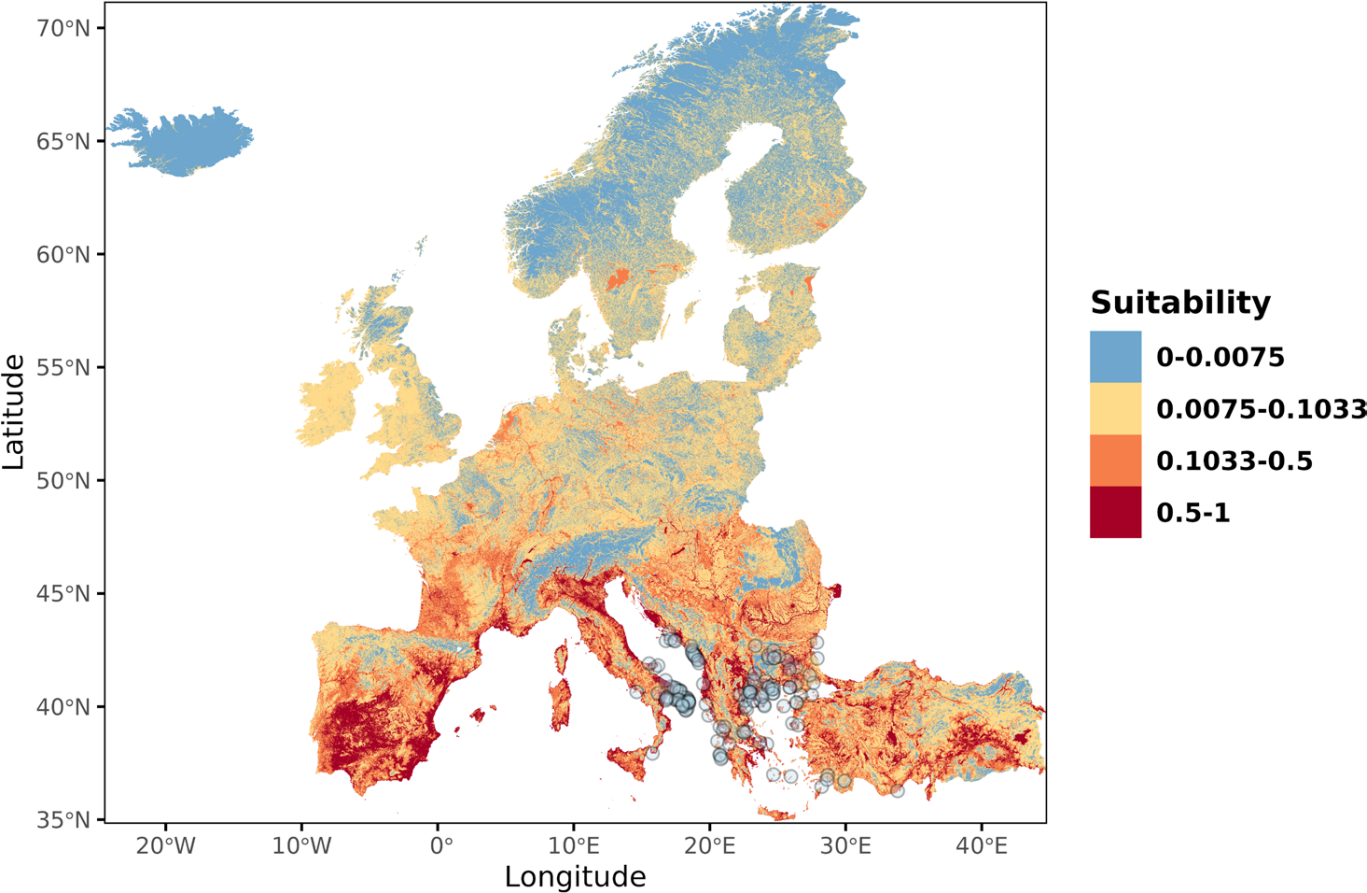
Predicted habitat suitability for the giant water bug *Lethocerus patruelis* across Europe, based on a fine‐tuned Maxent species distribution model. Habitat suitability values range from 0 (unsuitable) to 1 (highly suitable). The minimum training presence threshold is 0.0075, and the 10th‐percentile training presence threshold is 0.1033. Observations are shown in light blue.

**FIGURE 5:**
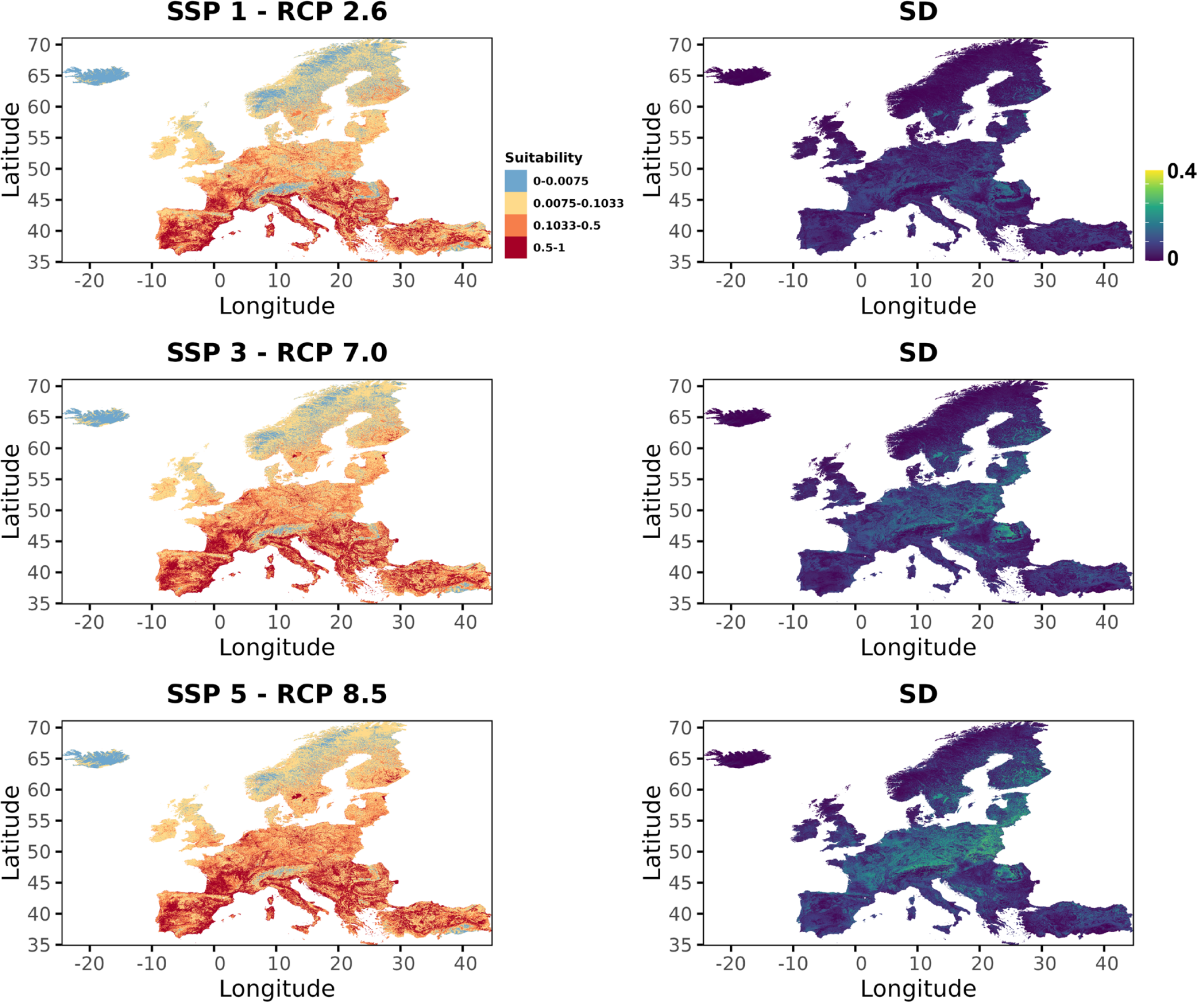
Predicted habitat suitability for *Lethocerus patruelis* in Europe for 2041–2070 under three climate scenarios: SSP1‐RCP2.6, SSP3‐RCP7.0, and SSP5‐RCP8.5. The right panel shows the standard deviation among five General Circulation Models (GCMs). Habitat suitability values range from 0 (unsuitable) to 1 (highly suitable). The minimum training presence threshold is 0.0075, and the 10th‐percentile threshold is 0.1033.

Concerning the SDM, all variables had VIF values below the recommended threshold of 3 (Data S1.4); thus, they were all included in the final model. The fine‐tuned Maxent model performed well (CBI = 0.914; AUC = 0.790), indicating robust predictive ability on the withheld dataset and including linear, quadratic, and hinge features, with a regularization multiplier of two (Data S1.5), and outperformed all alternative models in terms of both CBI and AUC. The most important variables, according to permutation importance, were cropland cover (44.8%), precipitation seasonality (30.3%), and temperature of the warmest quarter (17.1%; Data S1.6). Response curves for the two climatic variables showed preferences for values typical of the Mediterranean region, while cropland cover was negatively associated with suitability (Figure 3). Other variables had only a marginal influence (tree cover: 4.1%; freshwater cover: 3.7%; Data S1.6). Spatial projections for the current period highlighted large potentially suitable but unoccupied areas, particularly in Italy and other Mediterranean countries (Figure 4). Projections under future climate change scenarios (2041–2070) consistently indicated an expansion of suitable areas across Europe, especially under the most severe scenarios, with a clear northward shift (Figure 5). Model agreement among GCMs was generally high (SD of habitat suitability across five GCMs always < 0.4; Figure 5), although uncertainty increased under the most extreme scenario (Figure 5).

The model excluding the easternmost observations assigned higher importance to precipitation seasonality (66.6%), followed by cropland cover (13.9%) and temperature of the warmest quarter (13.4%; Data S1.6). Nonetheless, projections under current and future conditions were largely consistent with those obtained from the full dataset (Figures S2.2 and S2.3).

## 4 Discussion

Despite the lack of evidence for established breeding populations, the frequency of recent records of *Lethocerus patruelis* far from the Adriatic coast suggests that observations are unlikely to represent sporadic arrivals from the Balkans. Instead, the directional expansion we observed indicates an ongoing colonization process. Westward expansion can be readily explained by the fact that the earliest Italian records of the giant water bug were located near the Adriatic coast (Cianferoni and Nardi 2013), making this directional trend largely constrained by geography. By contrast, the southward expansion observed in Italy differs from patterns reported in other countries, such as Bulgaria, where the species has been documented to expand northward (Grozeva et al. 2013).

Analyses of niche dynamics suggest that the expansion is still ongoing, as high levels of niche unfilling are typical of range‐expanding species during the colonization phase (Petitpierre, et al. 2012; Strubbe et al. 2013). Niche expansion is the only measure reflecting “true” niche shifts (Petitpierre, et al. 2012; Strubbe et al. 2013; Li et al. 2014; Liu et al. 2020), thus our results indicate that *L. patruelis* niche is conserved in the novel range. This supports the reliability of SDM projections outside the training area (Early and Sax 2014; Pili et al. 2020; Atwater and Barney 2021).

The results of SDM under current environmental conditions confirm the species' preference for Mediterranean conditions, with the highest levels of suitability found around the Mediterranean basin. Climate change has been proposed as a likely driver of *L. patruelis* spread in Italy (Cianferoni and Mazza 2023), but this study is the first to investigate the relationship between climatic factors and the species' potential distribution. Contrary to expectations, suitability was mostly affected by cultivated surfaces, showing higher suitability in areas with low agricultural cover. While Belostomatidae can exploit traditionally managed rice fields (Christopoulos et al. 2022; Ohba 2019), they thrive in complex landscapes where agricultural lands are integrated with irrigation and drainage channels, forested areas, and other semi‐natural habitats (Ohba 2019). In European Mediterranean countries, heavily cultivated areas often consist of monocultures, both woody (olive orchards, vineyards) and herbaceous (durum wheat; Franco et al. 2022; Lago‐Olveira et al. 2023; Zimmerer et al. 2024), which may explain the negative relationship between suitability and cropland in our model.

Counter‐intuitively, freshwater cover did not affect suitability, despite the biology of *L. patruelis* . This may be because nymphs are most often found in temporary shallow waterbodies (Nesemann and Sharma 2013), which are difficult to detect in remote‐sensing datasets. Another factor is that most of our observations are adults, which often display migratory behavior in response to seasonal conditions or food shortages (Ohba 2019). Adult migration may decouple observations from local waterbody availability, masking the importance of freshwater cover in the models at the selected spatial scale.

Based on our results, the Apennine chain may constrain the expansion of the giant water bug in Italy, particularly toward the northwest, as suitability was generally low in mountainous areas. This may explain the observed southward expansion, as *L. patruelis* seems to favor low‐altitude habitats, such as river deltas and slow‐moving streams close to sea level (Christopoulos et al. 2022). Future SDM projections suggest an increase in suitable areas, particularly in Central Europe and along Mediterranean mountain ranges such as the Apennines and the Pyrenees. The projected expansion of *L. patruelis* resembles patterns reported for other European hemipterans: many Mediterranean‐adapted species are expanding their ranges in response to global change, often through uphill shifts and/or northward expansions (Musolin and Fujisaki 2006; Musolin 2007; Rabitsch 2008). Traditionally, mountain chains acted as barriers limiting the northward spread of Mediterranean species, but climate change, land‐use change, and human activities are increasingly reducing their effectiveness (Rabitsch 2008). Our results are consistent with this trend, suggesting that the spread of the giant water bug in Europe may be facilitated by ongoing climatic changes.

### 4.1 Implications for Biodiversity Conservation and Invasive Species Ecology

Colonization of new areas by expanding species can disrupt local community structure, food webs, and functional diversity (Ficetola et al. 2012; Collins et al. 2016; Hoffmann and Courchamp 2016). The ongoing range expansion of *L. patruelis* in Italy highlights potential ecological consequences for freshwater ecosystems. Giant water bugs are top invertebrate predators, and their establishment in novel habitats may alter trophic dynamics of freshwater communities. Although evidence of ecological impacts outside the species' native range is limited, the combination of climate change and the arrival of new predators is a recognized driver of local extinctions and altered food webs in freshwater ecosystems (Ficetola et al. 2012; Harvey et al. 2023). Monitoring this expansion is therefore critical for early detection and management of potential impacts on aquatic biodiversity.

### 4.2 Limitations and Future Perspectives

Despite the insights provided by this study, several limitations should be acknowledged, which also suggest directions for future research. For example, the heterogeneity of data sources may have introduced biases into our dataset. Most retained observations come from Europe, likely reflecting a higher number of contributors rather than true local abundance. Although we accounted for geographical biases in the Maxent models, this may have led to an underestimation of the giant water bug's niche in its historical range. Additional limitations concern the choice of predictors in niche analyses and SDMs. Temporal mismatches between occurrence records and climatic or land‐use data, while unavoidable due to the lack of better alternatives, may introduce some uncertainty. Furthermore, although high‐resolution future climate projections are readily available, equivalent datasets for LULC are lacking, forcing us to assume static land‐use conditions in our models. Future research should aim to address these limitations to validate and refine the findings of this study. Targeted field surveys are needed to obtain direct evidence of naturalization of the giant water bug in Italy, such as the presence of established breeding populations.

## 5 Conclusions

Our findings indicate that *L. patruelis* is undergoing a directional range expansion in Italy, likely facilitated by ongoing climate change. This expansion occurs without detectable niche shifts, demonstrating strong niche conservatism. Species distribution models reveal large climatically suitable but currently uncolonized areas across Europe, with projected suitability expected to increase further under pessimistic climate scenarios. The observed combination of spatial expansion and niche conservatism suggests that the species has not yet reached its climatic limits, highlighting the importance of continued monitoring. As a top aquatic predator, *L. patruelis* has the potential to alter freshwater trophic dynamics, particularly in temporary or anthropogenically modified habitats. Although direct evidence of ecological impacts outside its native range remains limited, the interplay of climate change and invasive predators is a well‐recognized driver of local extinctions in freshwater ecosystems. Further research into the species' biology, dispersal mechanisms, and ecological impacts will be crucial to anticipate its spread and to inform conservation and management strategies for vulnerable freshwater communities.

## Author Contributions

**Andrea Simoncini:** conceptualization (equal), formal analysis (lead), methodology (lead), visualization (lead), writing – original draft (equal), writing – review and editing (equal). **Filippo Tomasi:** conceptualization (equal), data curation (equal), investigation (equal), writing – review and editing (equal). **Gentile Francesco Ficetola:** conceptualization (equal), methodology (supporting), supervision (lead), writing – review and editing (equal). **Elia Lo Parrino:** conceptualization (equal), data curation (equal), formal analysis (supporting), investigation (equal), methodology (supporting), supervision (supporting), writing – original draft (equal), writing – review and editing (equal).

## Conflicts of Interest

The authors declare no conflicts of interest.

## Supporting information


**Data S1:** ece372458‐sup‐0001‐DataS1.xlsx.

**Data S2:** ece372458‐sup‐0002‐DataS2.docx.

**Data S3:** ece372458‐sup‐0003‐DataS3.docx.

## Data Availability

The datasets and R code used in this study are available from: https://zenodo.org/records/14046410.
