# Supplementary material for: Range Expansion of the Giant Water Bug Lethocerus patruelis (Stål, 1854) in Europe
Source: Ecol Evol. 2025 Nov 12;15(11):e72458. doi: 10.1002/ece3.72458 (PMC12606002; doi:10.1002/ece3.72458)
Supplement: Supplementary file 2 — Data S2: ece372458‐sup‐0002‐DataS2.docx. [file ECE3-15-e72458-s001.docx]

# Supporting Information

# DataS1


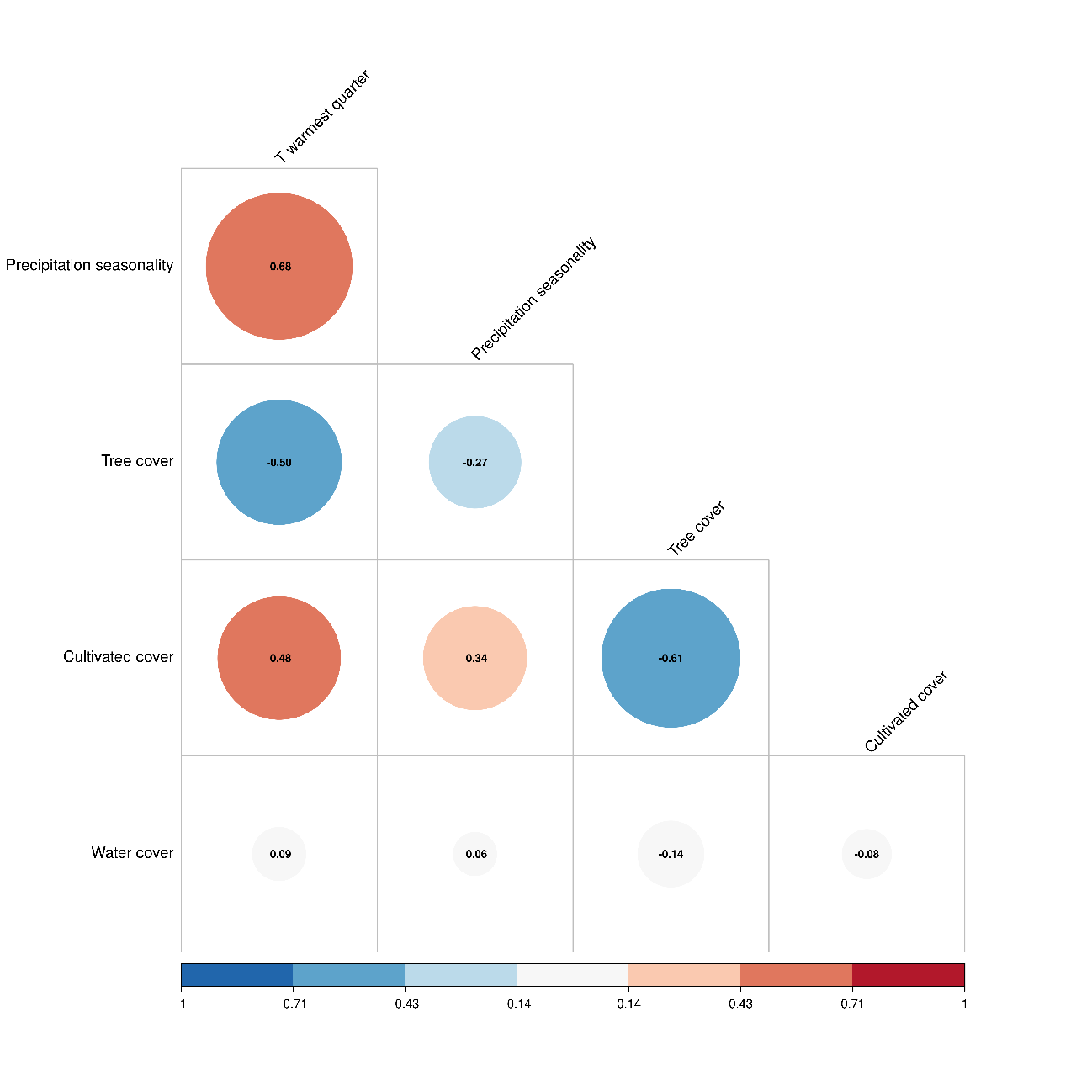
**Figure S2.1:** Pairwise Pearson’s correlation among predictor variables employed for modelling the ecological niche and the expansion of the giant water bug *Lethocerus patruelis*. Values are computed in a buffer of 100 km around all occurrence points used for models.
**Figure S2.2:** Predicted habitat suitability for the giant water bug *Lethocerus patruelis* across Europe, based on a fine-tuned Maxent model (reduced dataset). Suitability ranges from 0 (unsuitable) to 1 (highly suitable). Minimum training presence threshold = 0.03; 10th-percentile training presence threshold = 0.2043. Observed occurrences are shown in light blue.
**
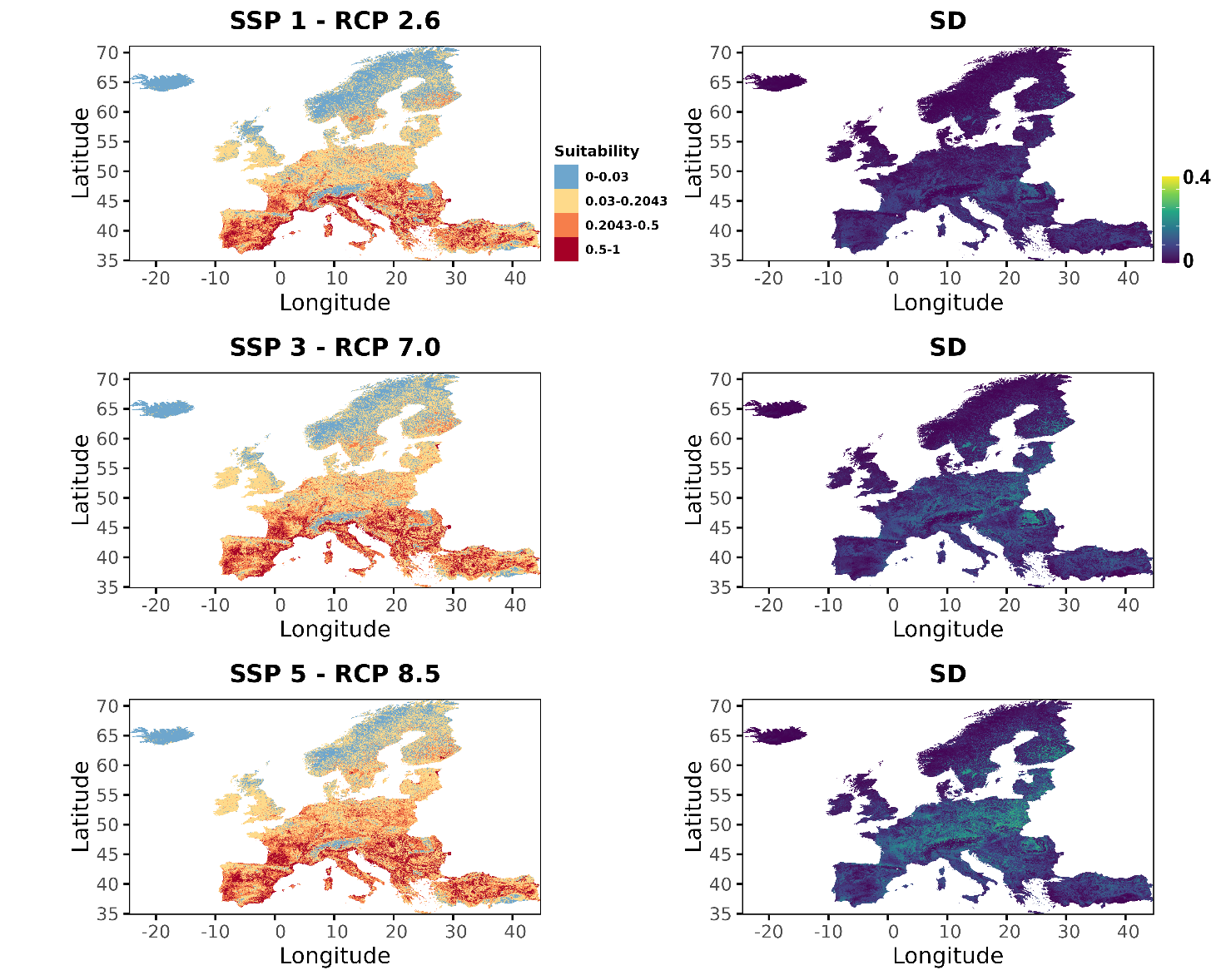
 Figure S2.3.** Predicted habitat suitability for *Lethocerus patruelis* in Europe (2041–2070) under three climate scenarios (SSP1-RCP2.6, SSP3-RCP7.0, SSP5-RCP8.5), based on the reduced-dataset model. The right panel shows the standard deviation among five GCMs. Suitability ranges from 0 to 1, with thresholds as in Fig. S2.2.
